# Swimming behaviour tunes fish polarization vision to double prey sighting distance

**DOI:** 10.1038/s41598-018-37632-1

**Published:** 2019-01-30

**Authors:** Iñigo Novales Flamarique

**Affiliations:** 1 Department of Biological Sciences, Simon Fraser University, Burnaby, British Columbia V3J 4M5 Canada; 2 Department of Biology, University of Victoria, Victoria, British Columbia V8W 2Y2 Canada

## Abstract

The analysis of the polarization of light expands vision beyond the realm of colour and intensity and is used for multiple ecological purposes among invertebrates including orientation, object recognition, and communication. How vertebrates use polarization vision as part of natural behaviours is widely unknown. In this study, I tested the hypothesis that polarization vision improves the detection of zooplankton prey by the northern anchovy, *Engraulis mordax*, the only vertebrate with a demonstrated photoreceptor basis explaining its polarization sensitivity. Juvenile anchovies were recorded free foraging on zooplankton under downwelling light fields of varying percent polarization (98%, 67%, 19%, and 0% - unpolarized light). Analyses of prey attack sequences showed that anchovies swam in the horizontal plane perpendicular, on average, to the polarization direction of downwelling light and attacked prey at pitch angles that maximized polarization contrast perception of prey by the ventro-temporal retina, the area devoted to polarization vision in this animal. Consequently, the mean prey location distance under polarized light was up to 2.1 times that under unpolarized conditions. All indicators of polarization vision mediated foraging were present under 19% polarization, which is within the polarization range commonly found in nature during daylight hours. These results demonstrate: (i) the first use of oriented swimming for enhancing polarization contrast detection of prey, (ii) its relevance to improved foraging under available light cues in nature, and (iii) an increase in target detection distance that is only matched by polarization based artificial systems.

## Introduction

Besides colour and intensity, light has another physical attribute that some animal visual systems can detect termed its polarization^1^. The polarization of light refers to the dominant electric field direction of the ensemble of photons that comprise it. If all the photons from a light source oscillate in the same plane, light is termed 100% linearly polarized in that plane (the E-vector plane). In contrast, light without a preferred plane of electric field oscillation, like that emitted by the sun, is unpolarized or 0% polarized. Unpolarized light becomes partially polarized in multiple ways including scattering by particles in the atmosphere and water and reflections from smooth surfaces^2^. This provides additional visual cues that can be used to improve the contrast of targets or for orientation and navigation, as is the case for various invertebrates^2,3^.

As opposed to the retinal photoreceptors found in invertebrates, which differentially absorb naturally incident light on the retina as a function of polarization^2–4^, a phenomenon known as axial dichroism, those of vertebrates have no equivalent preferred axial absorbance. As such, polarization sensitivity in vertebrates, except in a group of anchovies in the family Engraulididae^5–9^, does not have a demonstrated mechanistic foundation at the photoreceptor level and is highly controversial^10^. An exception to the lack of axial dichroism of vertebrate photoreceptors is found in the retinas of anchovies^11^. These animals have unique cone photoreceptors in that their outer segments consist of lamellae (i.e., the lipid bilayers which house the visual pigments) parallel to their lengths^5–9^. Because absorbance is restricted to the plane of the lamellae^11^, these photoreceptors are axially dichroic.

**Figure 1:**
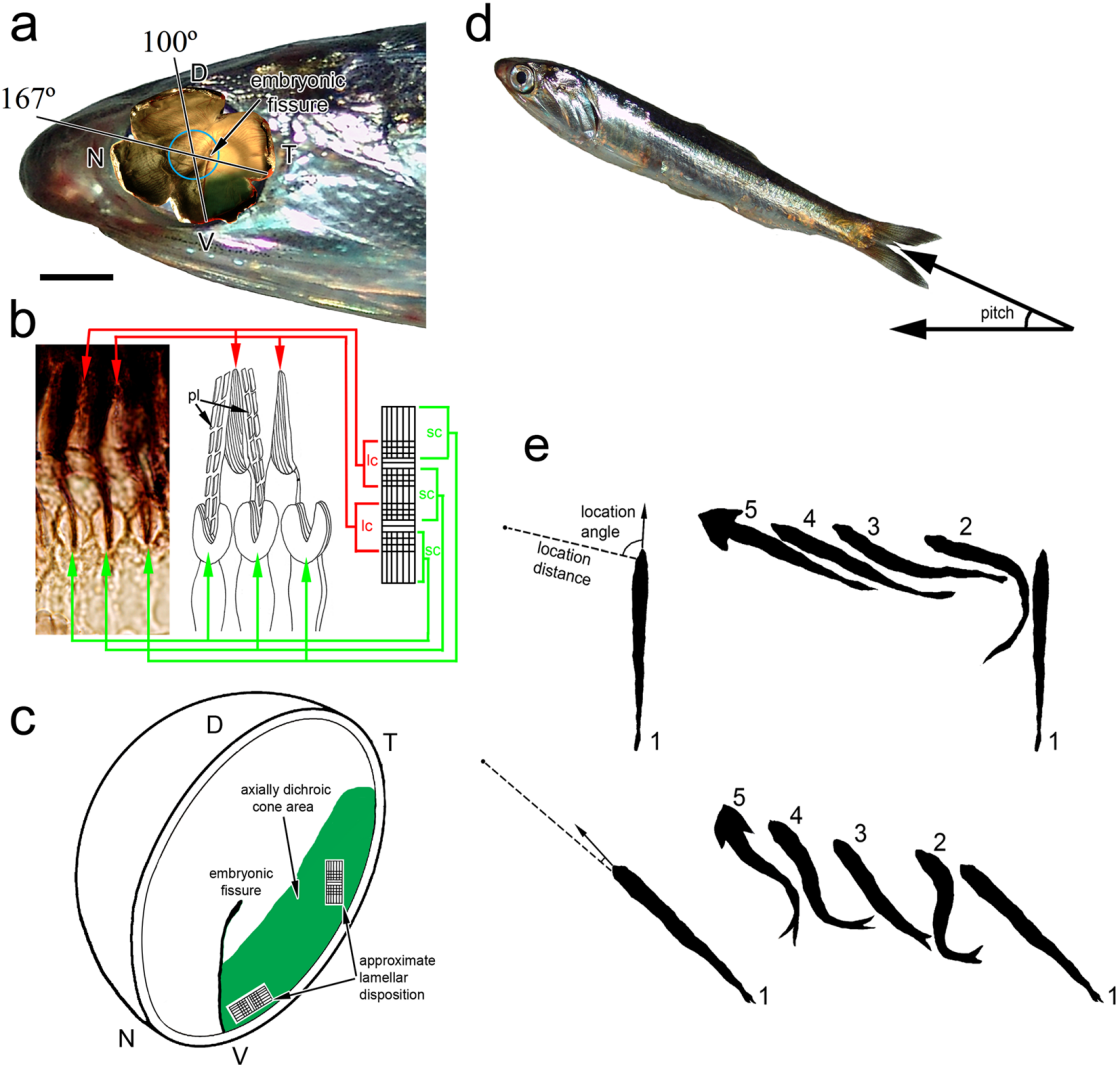
Characteristics of the anchovy retina and illustrations of prey capture behaviour and measured variables. (**a**) Retinal flat mount superimposed on the head of a northern anchovy showing the polarization-sensitive area in the ventro-temporal retina with axially dichroic cones. This area appears green due to reflectance by stacks of plates associated with the outer segments of the long cones. The blue circle depicts the location of the lens. The arrow points to the embryonic fissure, which extends from the ventral periphery to the centre of the retina (location of the optic nerve head) and points toward the temporal retina. In this (typical) retina, the field of view associated with the polarization sensitive area expands 100–167° of the trigonometric circle (or 13–80° from the horizontal). (**b**) Radial cryosection showing the disposition of the axially dichroic cones and associated diagrams illustrating the orientation of the lamellae in the long cone (lc) and short cone (sc). The long cone has a cuneate outer segment flanked by stacks of platelets (pl) on the ventral and dorsal sides (one such set is shown). Each long cone inner segment is squeezed between opposite lobes of adjacent bilobed outer segments of two short cones. The rightmost diagram shows the disposition of lamellae looking down on the retina. In the area of highest cone density, the long cone lamellae are approximately parallel to the horizontal whereas those of the short cone are oriented vertically. The cross-hatched areas denote regions of overlap between the two outer segment types. (**c**) Schematic of an eye-cup showing the approximate region of the ventro-temporal retina with axially dichroic cones (in green) and the disposition of the lamellae of a long cone with associated flanking short cones [same depiction as in (**b**)] in two areas of the retina. (**d**) Northern anchovy illustrating the pitch (elevation) angle. (**e**) Two prey capture silhouette sequences obtained under unpolarized light (top) and 98% polarization (bottom). In each sequence, the leftmost silhouette (1) shows the fish immediately prior to attack initiation with associated location distance (dash line uniting the fish to the prey, the latter represented as a dot) before correction for pitch angle, and location angle. The associated diagrams (1–5) on the right are postures of the fish, every 0.2 seconds, after attack initiation. Silhouette 5 shows opercular expansion at the moment of prey capture. Silhouette 4 from the top sequence and 3 from the bottom sequence are representative of those used to estimate pitch angle. The magnification bar in (**a**) is 1.5 mm, 3.3 µm for the section in (**b**), and 1 cm in (**d**). Abbreviations: N, nasal; D, dorsal; V, ventral; T, temporal directions.

Two types of cone, a long cone with cuneate outer segment and a short cone with bilobed outer segment, alternate with orthogonal lamellar disposition between them forming axially dichroic rows that are primarily restricted to the ventro-temporal retina in the northern anchovy and other anchovy species (Fig. 1)^7–9^. Other areas of the retina in the northern anchovy have cones with regular lamellar disposition, i.e., transverse to the length of the cell^12^. The two types of axially dichroic cones house a middle wavelength sensitive visual pigment with statistically similar wavelength of maximum absorbance (λ_max_)^10,13^. The remainder of the retina has at least two visual pigments among the cone population with λ_max_ in the middle and long wavelength regions of the spectrum^10,13^.

In the northern anchovy, *Engraulis mordax*, optic nerve recordings show that the ventro-temporal area of the retina comprising axially dichroic cones exhibits polarization sensitivity but lacks colour sensitivity, as judged by a single peak, at 520 nm, spectral sensitivity function^10^. The rest of the retina shows a two peak spectral sensitivity function, at 500 nm and 540 nm, suggesting colour discrimination^10,14^. The spectral sensitivity findings are in accord with the number of visual pigments and the distribution of opsin transcripts in the retina^14^. In essence, the retina of the northern anchovy is structurally and functionally divided for polarization and colour vision in analogy with the retina of multiple insect species and stomatopods^2^.

In a previous investigation^10^, the northern anchovy was observed to discriminate between a wide angle 100% linearly polarized downwelling light field and an unpolarized (0% polarized) light field of the same intensity and spectral composition. Furthermore, anchovies changed their swimming behaviour from horizontal when under unpolarized light, to oblique loops at pitch angles above the horizontal when under 100% polarization, and the anchovy was often oriented with its body perpendicular to the downwelling polarization. These observations gave rise to the hypothesis that the northern anchovy may be orienting its body axis to maximize sensitivity to the background polarization by preferential stimulation of the long cones^8,10^, thus improving polarization contrast vision of targets in the upper frontal field, the area viewed by the polarization sensitive ventro-temporal retina (Fig. 1)^8,10,15^. Here, this hypothesis was tested by examining the behaviour of juvenile anchovies free foraging on saltwater tolerant *Daphnia pulex*, a translucent zooplankton prey.

Based on the location and extent of the polarization-sensitive area in the northern anchovy retina and the orientation of lamellae of the axially dichroic cones^8^, a series of predictions could be advanced to test the hypothesis of oriented swimming for improved polarization contrast of prey. First, within the area occupied by axially dichroic cones (Fig. 1a), the region of greatest cone density is concentrated in the mid to peripheral ventro-temporal retina^8^ viewing an upper frontal field around 30°–60° in elevation (pitch) from the horizontal and about 0–45° in azimuth from the forward direction. Thus, if high resolution polarization contrast vision were used by the anchovy to focus on prey, the pitch angle of the body should be within the 30–60° range during the strike phase. In contrast, this angle should be generally lower, approximating horizontal attacks, under unpolarized conditions, when the visual advantage conferred by axially dichroic cones would, theoretically, diminish. The horizontal (azimuth) angle of attack should in general be greater under unpolarized *versus* polarized conditions given the limited azimuth viewing angle (~0–45°) associated with the axially dichroic cone area. Non-axially dichroic cones are present in the upper third of the ventro-temporal retina (Fig. 1a,c) providing the polarization independent photoreceptor input looking forward and sideways.

Second, in the area with the highest density of axially dichroic cones, the lamellae of the long cones are approximately aligned with the horizontal direction whereas those of the short cones are aligned with the vertical direction^8,10^ (Fig. 1b,c). Because the retina is hemi-spherical, the lamellae of the long cones would primarily be oriented perpendicular to the fish length whereas those of the short cones would approach the vertical toward the mid-retina and above, and align with the length of the fish toward the lower ventral retina (Fig. 1c). Thus, if swimming parallel to the water surface, maximum sensitivity by the ventral retina to a downwelling polarized light field could be achieved by swimming perpendicular to the polarization (E-vector) direction, preferentially activating the long cones, or by swimming parallel to the E-vector, preferentially activating the short cones. If the polarized background were primarily in the forward direction, however, maximum sensitivity to the background polarization would involve long cone activation, as the fish would need to swim sideways in order to preferentially activate the short cones under this optical scenario. Swimming sideways is only associated with loss of the upside reflex in dying anchovies (personal observation). As such, for natural behaviours, overall maximum sensitivity to the background polarization could be achieved by swimming perpendicular to the E-vector.

Third, if polarization vision improved detection of prey by the northern anchovy, then the mean distance at which the animal locates prey, termed the location distance^16^, should be significantly greater under polarized *versus* unpolarized conditions. This is because zooplankton prey have chitineous exoskeletons that transform the polarization of background light via scattering^17^ and birefringence^16^, thereby enhancing their contrast to a polarization-sensitive predator. Polarization sensitivity should therefore give an ecological advantage to the anchovy in enhanced foraging. Analysis of the above variables [i.e., body axis orientation in the horizontal plane with respect to E-vector, pitch angle of attack, horizontal (azimuth) angle of attack, and location distance] should therefore reveal the polarization percentage that the anchovy can detect and use for enhanced prey contrast. In surface waters inhabited by anchovies, percent polarization is commonly in the range 10–30% during daylight hours^17,18^, depending on cloud cover, and can reach up to 67% during clear sky crepuscular periods^18^. Thus, any use of polarization vision for foraging during daylight hours would require a threshold detection level below 30%. In summary, this study tested whether oriented swimming constitutes a novel means of dynamically enhancing polarization contrast vision of prey by a vertebrate and its relevance to natural behaviours.

## Results

**Figure 2:**
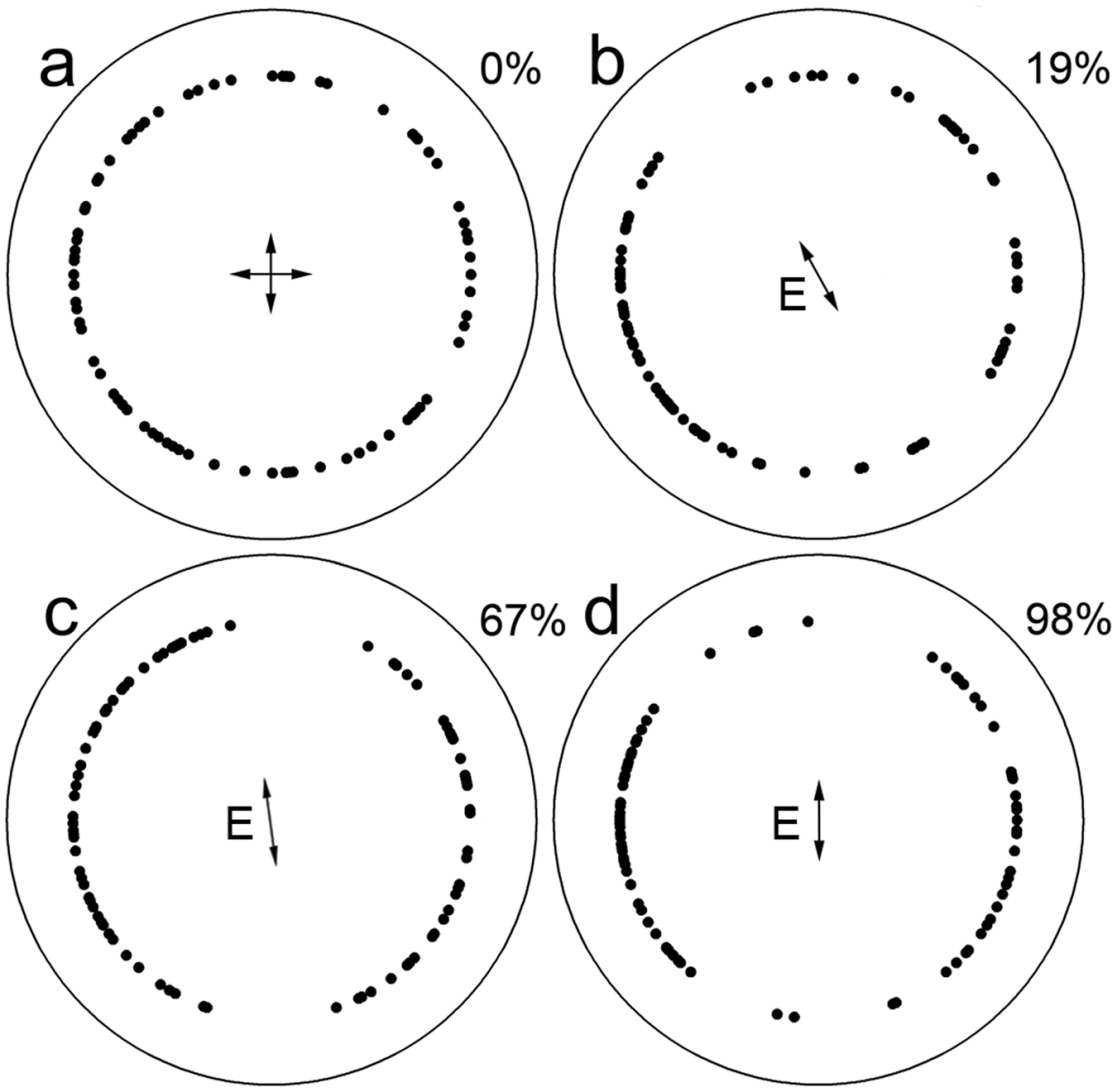
Angular orientation of northern anchovy to different percent polarizations. The measured E-vector (E_max_) bearing (shown as E) and percent polarization are indicated on each panel. The 0% polarization is indicated as crossed E-vectors. See Table 1 for statistics of distributions.

**Figure 3:**
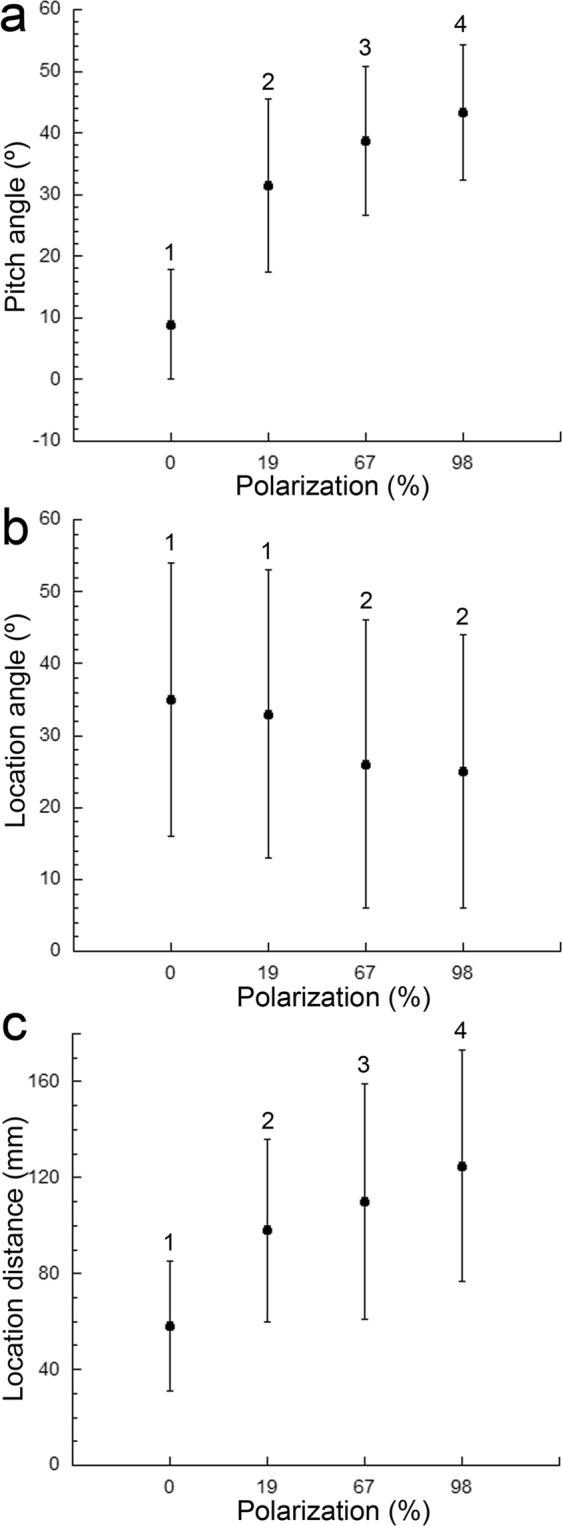
Mean (±SD) of the prey attack variables measured as a function of percent polarization. (**a**) Pitch (elevation) angle. (**b**) Location (azimuth or horizontal) angle. (**c**) Location distance. The ANOVA statistics were as follows: F_3,399_ = 150.1, p < 0.0001 (pitch angle); F_3,399_ = 6.437, p < 0.0001 (location angle); F_3,399_ = 46.14, p < 0.0001 (location distance). In each graph, means designated with different numbers are statistically different.

Under unpolarized light, anchovies swam randomly, on average, immediately prior to initiating attacks (Fig. 2a, Table 1). In contrast, under the various polarizations tested (19%, 67% and 98%), swimming prior to attack initiation was not random but with mean orientation perpendicular to the E-vector (Fig. 2b–d, Table 1). Thus, anchovies aligned their body axes for maximum stimulation of the long cone lamellae by the downwelling polarization (Fig. 1a,b). Analysis of the pitch (elevation) angle (Fig. 1c) associated with the attacks further reinforced this conclusion as the mean angle under 98% polarization was 43° (Fig. 3a), which approached the median (45° ± 4°, n = 5) of the angular range associated with the axially dichroic cone area (Fig. 1a). As the percent polarization decreased, so did the mean pitch angle of attack, but even under 19% polarization, it was significantly greater than under 0% polarization (Fig. 3a).

**Table 1:** Statistical results of Rayleigh’s test applied to northern anchovy double swimming angles.

Corresponding figure	% polarization	ā (°)	SD	a (°)	R	z	u
2a	0	178	101	none	11.384	1.296	N/A
2b	19	210	78	218	23.156	5.362	3.229
2c	67	193	95	190	28.765	8.274	4.052
2d	98	185	105	180	28.956	8.384	4.086

Symbols are as follows: ā, mean double angle; SD, standard deviation; a, theoretical double angle; R, Rayleigh’s statistic; z, statistic for circular uniformity; u, statistic for distribution along a mean direction. The data represented by the mean double angle are uniformly distributed around the trigonometric circle if z ≤ z_0.05,100_ = 2.988. The data are distributed along a specific mean direction (the angle a, corresponding to E_min_) if u ≥ u_0.05,100_ = 1.645. The panels on Fig. 2 corresponding to each statistic are indicated on the Table.

The azimuth, or horizontal, prey location angle (Fig. 1e) was on average lower for the highest polarizations (98% and 67%) compared to the 19% polarization and to unpolarized light (Fig. 3b). This may indicate the use of the upper third of the ventro-temporal retina, which is populated by non-axially dichroic cones, when foraging under low percent polarizations.

The mean location distance of prey (Fig. 1e) was 1.7, 1.9, and 2.1 times greater under 19%, 67% and 98% polarization, respectively, compared to that under unpolarized light, which had a mean of 58.6 mm (Fig. 3c). These results reveal an unprecedented improvement (>200%) in sighting distance of targets due to polarization vision.

## Discussion

The increase in location distance of targets achieved by the northern anchovy under the various downwelling polarization fields far exceeds similar measures reported in the literature for other animals. Squid hatchlings, for instance, improved their location distance of zooplankton prey by an average of 70% under 100% polarization over that under 0% polarization^19^ whereas the same comparison led to mean increases of 22% and 13% for juvenile rainbow trout^16^ and marine stickleback^20^, respectively. For fiddler crabs, mean response distance to an approaching target increased by 17% or 24% depending on whether the target was unpolarized or vertically polarized, respectively, with respect to a horizontally polarized target, over a natural background which was 30–50% horizontally polarized^21^. It is only for machine polarization visual systems that a comparable 2–3 fold increase in location distance of targets has been documented^22^.

The northern anchovy is the first species reported to exhibit oriented swimming for the purpose of enhancing polarization-based contrast detection of prey. This novel behaviour finds its functional equivalent in the rotational movements of stomatopod eyes^23^. These crustaceans align the microvilli of two sets of photoreceptors with orthogonal polarization sensitivity to maximize the contrast of the visual scene^23^. The anchovy achieves the same feat but by re-orienting its body. This is accomplished by swimming in a direction that aligns the lamellae of the long cones with the prevailing polarization, maximizing the contrast of objects that disrupt the background polarization. Body realignments but for the purpose of orientation to polarization patterns in the sky are also carried out by multiple insect species^3,24,25^.

The low threshold percentage (≤19%) for polarization use by the northern anchovy is also unprecedented among vertebrates. In fishes, the percent polarization threshold has only been estimated for juvenile rainbow trout and is between 63% and 72%^16,18^. Initial reports of polarization sensitivity of freshwater sunfishes have not been replicated^26^ and, with the exception of *Zenarchopterus dispar*^27^, other fish species have shown either no polarization responses^28^ or only to polarizations ≥ 95%^20,29^, which far exceed the available cues in nature^17,18^. The percent polarization threshold of the anchovy is in the range reported for crustaceans (15–30%)^30,31^, locusts (~30%)^32^, and other insects (5–10%)^24,33^.

As has been reported for insect interneurons^2,34^, the polarization signal at the level of the optic nerve in anchovies is the result of an antagonistic interaction between two photoreceptor channels (cone mechanisms) with orthogonal polarization sensitivity^10^. The net effect of this interaction is an increase in the gain of the differential signal between the two cone mechanisms^10^. In addition, light capture by the long cone is to some extent pre-filtered by the outer segments of the two flanking short cones (Fig. 1b)^8,11^. Capture of the transmitted horizontal polarization is then enhanced by plate stacks on either side of the long cone outer segment^8^. These specializations result in strongest sensitivity to the background (horizontal) polarization^10^, sensed primarily by the long cones, and minimal interference of polarization signals between cone types. Such a filtering scenario has been demonstrated to enhance the polarization sensitivity of the cell receiving the filtered light in invertebrate photoreceptors^4^. Any disturbance to the horizontal polarization of the water background, like that created by zooplankton prey, would therefore be readily detected by the anchovy.

The illumination set-up in the present study mimicked the downwelling direction under crepuscular periods when percent polarization is at its maximum in nature^18^. In this study, the *Daphnia* would have scattered light and appeared slightly darker against the downwelling background to the anchovy, and this contrast increased when the light was polarized *via* oriented swimming, as shown by the results. When viewing prey horizontally, however, prey contrast would have arisen primarily from the scattered downwelling light against the darker horizontal background with polarization playing little to no role in the modulation of perceived contrast. In nature, juvenile anchovies are commonly found feeding in surface waters during daylight hours. In these conditions, prey can be imaged against a lower intensity, sidewelling background that is predominantly horizontally polarized^17,18^. This would also allow the anchovy to maximize prey contrast using polarization but, in this case, scattered downwelling light would make the zooplankton appear brighter against the background^17^. In summary, the northern anchovy should be able to use polarization vision for improved contrast of zooplankton, and therefore achieve enhanced foraging^16^, throughout the day. This would give the anchovy a competitive advantage when foraging with other zooplanktivorous fishes, like herring and sardine, in detecting prey at greater distances and facilitating prey selectivity. There is, indeed, increasing evidence of trophic partitioning among such mixed fish schools with anchovies often performing selective feeding on larger taxa (e.g., cladocerans, copepods, and euphausiids) while sardines and herring carry out filter-feeding and capture smaller, less nutritious prey^35,36^.

## Materials and Methods

### Animals

Wild, young-of-the-year northern anchovy schools were caught in surface waters (<10 m depth) using a boat equipped with a purse seine net by staff from the Washington Department of Fish and Wildlife and the US Geological Survey (Marrowstone Marine Station) and by staff from Westport Seafood Ltd (Washington State, USA). The fish were then transported to the University of Victoria Aquatic Facility where they were held in a 5,000 l outdoor tank with circulating ocean water. The study took place during the month of August, and the fish experienced the natural daylight cycle throughout the study. Fish measuring 5.2 cm in total length were selected from the school for foraging experiments. It was important that there be no variation in length between fish as two individuals were used in each experiment and the pitch angle associated with each prey attack was derived from the length of the fish silhouette in the video (Fig. 1e) and the actual length of the fish using trigonometry. The fish were fed live *Daphnia pulex* for two weeks prior to the start of experiments. These *Daphnia* were the progeny of a population gathered in brackish waters near Victoria that had been selected for salt water tolerance for >5 years. All animal use was approved by the Animal Care committees of Simon Fraser University (protocol #1126B-10) and the University of Victoria (protocol #2017-005), which abide by regulations set by the Canadian Council for Animal Care.

### Imaging and illumination systems

Silhouette video photography was used to record northern anchovy free foraging on *Daphnia pulex* under a full spectrum downwelling light field that varied in percent polarization. The system was configured to record fish positions from above with a resolution of ~0.2 mm and a depth of field of 15 cm. Silhouettes of animals were achieved using a dim light emitting diode (LED) located below the aquarium at the focal point of a 20 cm diameter collimating lens whose output traversed the aquarium. The outermost walls of the aquarium were covered with black matte surface contact paper. The upwelling LED emission at the level of the bottom surface of the aquarium was below the detection threshold (10^12^ photons m^−2^ s^−1^) of a USB-2000 spectroradiometer equipped with a UV-visible liquid light guide (0.22 NA) and cosine collector (Ocean Optics). Projected silhouettes of the fish and prey within the field of view (a 20 cm diameter circle) were acquired with a camera situated above the aquarium. The field of view was a minimum of 10 cm from the walls of the aquarium reducing the probability of edge effects; only animals swimming freely in the water column were imaged and their displacements analyzed.

The illumination consisted of a Fiber-lite MI-150 (Dolan-Jenner Industries) coupled to a UV-visible liquid light guide (Photon Technology International) equipped with a diffuser and lens at its end. This emission was unpolarized, as verified with irradiance measurements for different rotations of a polarizer placed after the lens. To achieve various percent polarizations (98%, 67%, 19%, and 0%) of the same intensity and spectral content (λ: 320–800 nm; irradiance: 8.12 × 10^14^ photons cm^−2^ s^−1^; see Supplementary Fig. S1 for sample spectral irradiance at E_max_ and E_min_ for the 19% polarization) a holder was fitted after the lens containing a linear polarizer (HNP’B, Polaroid) and a mica quarter wave plate (Melles Griot)^30^. Percent polarizations for various rotations of the quarter wave plate were calculated from irradiance measurements at the maximum (E_max_) and minimum (E_min_) planes of polarization, the latter determined visually at the level of the aquarium with an E-vector finder (Oriel). Percent polarization was computed as: Polarization (%) = 100 (I_Emax_ − I_Emin_)/(I_Emax_ + I_Emin_), where I_Emax_ and I_Emin_ were the irradiances in the E_max_ and E_min_ planes, respectively.

Because of the central positioning of the recording camera, the downwelling illumination was projected at an angle of 5° with respect to the vertical and was confined, quasi-uniformly, to the central 20 cm diameter circle of observation. Downwelling irradiance measurements on either half of the aquarium showed a difference of less than 1%. This difference was insignificant to the orientation behaviour of the anchovy, as indicated by the results obtained. Sidewelling irradiance measurements were below the detection threshold of the spectroradiometer, which was similar to that of the northern anchovy as assessed using optic nerve recordings^10^.

### Foraging experiments

Each experiment consisted of filming 2 new fish at a time foraging for 30 minutes in the 30 × 30 × 30 cm glass aquarium filled to a depth of 15 cm, after 30 minutes of fish acclimation. The fish were starved for 24 hrs prior to testing and experiments were performed during the day, i.e., during the light phase of the animal’s circadian rhythm. Prey concentration in all experiments was 5 l^−1^ and prey size (overall mean *Daphnia pulex* carapace length ± SD: 1.1 ± 0.18 mm, n = 150) was statistically equivalent between experiments.

Anchovies searched for prey using pause-travel movements whereby the fish combined stationary periods of scanning for prey with repositioning, swimming movements. Foraging behaviour was evaluated by measuring the following variables associated with each attack on prey (Fig. 1d,e): angle of the body axis with respect to the dominant polarization (E-vector, or E_max_); pitch (elevation) angle with respect to the horizontal; horizontal (azimuth) location angle of prey with respect to the fish; and prey location distance. The prey location angle was the angle between the longitudinal body axis of the fish just prior to attack initiation and the line connecting the fish’s rostrum and the position of the prey upon attack initiation (Fig. 1e). Prey location distance was defined as the distance between the point (fish rostrum) at which the fish first reacted to the prey and the position of the prey itself (Fig. 1e), but corrected for pitch angle. From the prey capture moment, characterized by fish opercular expansion (Fig. 1e), the video was back-tracked to the point when the fish first spotted the prey and initiated the attack. This moment was characterized by a quick change in the fish’s head direction, increased swimming velocity, and realignment of the body axis with the prey (Fig. 1e). The pitch angle was the angle between the body axis of the fish and the horizontal (Fig. 1d), assessed within the first 0.5 seconds of attack initiation. All attacks within this time period had frames showing alignment of the body axis from which an estimate of the pitch angle could be obtained using trigonometry given the fish length of 5.2 cm. The accuracy of this estimate was assessed to ±7° from trials where the walls of the aquarium were not blocked with matte contact paper and simultaneous filming was carried out with two additional cameras, positioned horizontally, at right angles to the aquarium. This potential error would change the computation of location distance by a maximum of 0.75%, which was insignificant to the statistics.

### Statistical analyses

A total of 100 prey attack sequences were analyzed from 5 replicate experiments per polarization condition. None of the attacks involved interference between anchovies so that each attack was considered an independent observation. Various statistical analyses were performed to detect any differences in the measured variables with polarization condition. Analysis of fish orientation with respect to the dominant E-vector (E_max_) involved Rayleigh’s test for circular uniformity. If the data were not uniformly distributed, a V-test was carried out to determine whether there was a preferred distribution in the E_min_ direction, i.e., perpendicular to E_max_^30^. The V test is a modification of the Rayleigh test that compares the mean directional angle of a non-uniform distribution to a single (theoretical) angle that is expected *a priori*^37^. Here, the expected angular direction was E_min_ based on activation of the long cones for maximal overall sensitivity of the fish to the background polarization. Differences in pitch angle, location angle, and location distance between polarizations were assessed by one-way Analysis of Variance (ANOVA) followed by Student-Neuman-Keuls and Tukey’s HSD post-hoc tests with α = 0.05.

### Animal experimentation approval

All animal use was approved by the Animal Care committees of Simon Fraser University (protocol #1126B-10) and the University of Victoria (protocol #2013-005) which abide by regulations set by the Canadian Council for Animal Care.

## Supplementary information

Supplementary Figure S1

## Data Availability

Supplementary Fig. S1 is included.
