# Supplementary material for: Swimming behaviour tunes fish polarization vision to double prey sighting distance
Source: Sci Rep. 2019 Jan 30;9:944. doi: 10.1038/s41598-018-37632-1 (PMC6353921; doi:10.1038/s41598-018-37632-1)
Supplement: Supplementary file 1 — Supplementary Figure S1 [file 41598_2018_37632_MOESM1_ESM.doc]

**Supplementary information**

**Article: Swimming behaviour tunes fish polarization vision to double prey sighting distance**

**Author: Iñigo Novales Flamarique**

**Supplementary Figure S1**

**Figure S1.** Spectral irradiance with the analyzer positioned in front of the spectroradiometer input and oriented at E_max_ or E_min_ for the case of 19% polarization.
